# Antibiotic Susceptibility, Biofilm Production, and Detection of *mec*A Gene among *Staphylococcus aureus* Isolates from Different Clinical Specimens

**DOI:** 10.3390/diseases9040080

**Published:** 2021-11-01

**Authors:** Upama Gaire, Upendra Thapa Shrestha, Sanjib Adhikari, Nabaraj Adhikari, Anup Bastola, Komal Raj Rijal, Prakash Ghimire, Megha Raj Banjara

**Affiliations:** 1 Central Department of Microbiology, Tribhuvan University, Kirtipur, Kathmandu 44600, Nepal; upaama.gaire@gmail.com (U.G.); upendrats@gmail.com (U.T.S.); sanadh26@gmail.com (S.A.); adhikarinaba13@gmail.com (N.A.); prakash.ghimire@cdmi.tu.edu.np (P.G.); 2 Sukraraj Tropical and Infectious Diseases Hospital, Teku, Kathmandu 44600, Nepal; docanup11@gmail.com

**Keywords:** *S. aureus*, antibiotic susceptibility, MRSA, *mec*A gene, biofilm

## Abstract

The increasing incidence of methicillin-resistant and biofilm-forming *S. aureus* isolates in hospital settings is a gruesome concern today. The main objectives of this study were to determine the burden of *S. aureus* in clinical samples, assess their antibiotic susceptibility pattern and detect biofilm formation and *mec*A gene in them. A total of 1968 different clinical specimens were processed to isolate *S. aureus* following standard microbiological procedures. Antibiotic susceptibility test of the isolates was performed by Kirby–Bauer disc-diffusion method following CLSI guidelines. Biofilm was detected through tissue culture plate method. Methicillin-resistant *S. aureus* (MRSA) isolates were screened using cefoxitin (30 µg) discs and *mec*A gene was amplified by conventional polymerase chain reaction (PCR). Of 177 bacterial growth, the prevalence of *S. aureus* was 15.3% (*n* = 27). MRSA were 55.6% (15/27) and 44% (12/27) exhibited multidrug resistance (MDR). There was no significant association between methicillin resistance and MDR (*p* > 0.05). Both MRSA and MSSA were least sensitive to penicillin (100%, 75%) followed by erythromycin (86.6%, 66.6%). Most of the MRSA (93.4%) were susceptible to tetracycline. All *S. aureus* isolates were biofilm producers—19 (70%) were weak and only one (4%) was a strong biofilm producer. The strong biofilm-producing MSSA was resistant to most of the antibiotics except cefoxitin and clindamycin. None of the MSSA possessed *mec*A gene while 8 (53.3%) MRSA had it. More than half of *S. aureus* isolated were MRSA. High incidence of multidrug resistance along with capacity to form biofilm among clinical isolates of *S.* *aureus* is a matter of apprehension and prompt adoption of biosafety measures is suggested to curb their dissemination in the hospital environments.

## 1. Introduction

*Staphylococcus aureus* is the most common bacteria associated with both community and hospital associated infections [1]. It is associated with several infections including wound infection, otitis media, osteomyelitis, recurrent urinary tract infection, endocarditis, and implant-related infections [2,3].

*S. aureus* can adhere to medical devices and form biofilm [4]. Biofilms are microbial-derived sessile communities embedded in a matrix of extracellular polymeric substances and bacteria growing in a biofilm are intrinsically more resistant to antimicrobial agents than planktonic cells [5]. Biofilm-forming methicillin-resistant *S. aureus* (MRSA) is particularly difficult to eradicate [6].

MRSA is one of the problematic bacteria since it alone results in almost half of all deaths caused by antibiotic resistant organisms [7]. Community-acquired MRSA strains often express lower levels of resistance to oxacillin and multiply faster than hospital-acquired MRSA strains with significantly shorter times which help community-acquired MRSA achieve successful colonization by enabling it to compete with the commensal bacterial flora [8]. Based on SCC*mec* typing, community-acquired MRSA strains are typically SCCmec IV, V, and VI, while hospital-acquired MRSA are usually SCC*mec* I, II, and III [9].

Methicillin resistance in *S. aureus* is mediated through an altered protein called low-affinity penicillin-binding protein (PBP2a). PBP2a is encoded by *mec*A gene and is present in chromosomal mobile genetic element called Staphylococcal cassette chromosome *mec* (SCC*mec*). Due to association of MRSA with multiple antibiotic resistance and higher cost of treatment, the accurate and rapid identification of MRSA is crucial in clinical setting for the management of MRSA infections. Continuous use of antibiotics in animal husbandry and agricultural activities can be one of the contributing factors for significant increase in resistance and transmission of *S. aureus* and MRSA [10].

In Nepal, the most common methods for detecting methicillin resistance are disc-diffusion with cefoxitin and oxacillin, with fewer reports on MIC determination and *mec*A gene identification by PCR [11]. In the past, various research in Nepal looked on the phenotypic prevalence of MRSA infections [12,13,14,15]. MRSA frequency has been found to range from 15 to 69% in different hospitals and laboratory settings in Nepal [13,14,15,16,17,18,19]. This study explores antimicrobial susceptibility patterns of clinical isolates of *S. aureus* from a tertiary hospital, examines their biofilm-forming capacity along with the detection of *mec*A gene.

## 2. Materials and Methods

### 2.1. Research Design

A cross-sectional study was conducted from September to March 2019 after acquiring ethical approval from the Institutional Review Committee of Institute of Science and Technology, Tribhuvan University. It was a qualitative, laboratory-based study.

### 2.2. Study Site and Specimens

All the specimens received at the Microbiology laboratory of Sukraraj Tropical and Infectious Disease Hospital, Kathmandu following physician’s order for investigative procedure were included in the study. The samples were collected as per standard microbiological techniques [20]. Samples obtained in a clean, leak-proof and screw-capped container with no visible signs of contamination and labeled properly with demographic information of patients were included in the study. Samples received from the patient under antiretroviral treatment were not included in the study. Molecular analysis of the samples was carried out at laboratory of Central Department of Microbiology, Tribhuvan University, Kirtipur, Kathmandu, Nepal.

### 2.3. Samples for the Study

A total of 1968 different clinical specimens including pus, urine, blood, sputum, urethral swab, CSF, and pleural fluid were collected at the Microbiology laboratory of the hospital and processed as per standard microbiological protocol for routine culture and antibiotic susceptibility testing [20,21].

### 2.4. Isolation and Identification of S. aureus

All the samples were inoculated directly onto blood agar and MacConkey agar. Suspected *S. aureus* colonies after Gram staining were inoculated on mannitol salt agar and incubated at 37 °C for 24 h. *S. aureus* colonies were identified based on colony characteristics on mannitol salt agar. Golden yellow colored, round, convex, opaque colonies were indicative of *S. aureus* and colonies were transferred to nutrient agar and incubated at 37 °C for 24 h. Furthermore, confirmation was done by biochemical tests including catalase, oxidase, DNase and coagulase tests [21].

### 2.5. Screening of Biofilm-Producing S. aureus

Screening of biofilm-producing *S. aureus* was done using tissue culture plate method (TCP). This quantitative test was performed as described elsewhere [22,23,24]. Briefly, a loopful of test organism was isolated from a fresh agar plate and inoculated in 2 mL of tryptic soya broth (TSB). The broth was incubated overnight at 37 °C. The culture was then diluted to 1:100 with fresh TSB medium. Individual well of 96 well microtiter plate was filled with 200 µL of diluted culture broths. The control organisms were also processed in a similar manner. The plate was incubated at 37 °C for 24 h. After incubation, the contents of each well were removed by gentle tapping. The wells were washed four times with 200 µL of phosphate buffer saline (pH 7.3) to remove free floating bacteria. Biofilms formed by bacteria adherent to the wells were fixed by 2% sodium acetate and then stained by 100 µL of 0.1% of crystal violet for 15 min at room temperature. Excess stain was washed gently with deionized water, and the plate was kept for drying. Biofilm was quantified by measuring the absorbance at 630 nm following solubilization of attached biofilm in 95% ethanol. The experiment was performed in triplicate and repeated three times. The interpretation of biofilm production was done according to the criteria mentioned elsewhere [25]. The criteria used were as follows: Non-producers = OD ≤ ODc, Weak biofilm producers = ODc ≤ 2ODc, Moderate biofilm producers = 2ODc < OD ≤ 4ODc, Strong biofilm producers = OD ≥ 4 ODc. The cut-off optical density (ODc) was defined as three standard deviations above the mean OD (optical density) of the negative control [22].

### 2.6. Antibiotic Susceptibility Test

Antibiotic susceptibility tests of all the isolates towards various antibiotics were performed by Kirby–Bauer disk diffusion method as recommended by Clinical Laboratory Standard Institute [26]. The turbidity of suspension on nutrient broth was compared with the turbidity standard of McFarland 0.5 and was uniformly carpeted on the surface of Mueller-Hinton agar using a sterile cotton swab. Then, antibiotics discs tetracycline, ciprofloxacin, gentamycin, clindamycin, cotrimoxazole, erythromycin and penicillin were placed. The plates were then incubated at 37 °C overnight. After incubation, the zone diameter organism was reported as resistant, intermediate or sensitive. Based on susceptibility pattern of the isolates, bacteria resistant to three or more than three classes of antibiotics were considered to be multidrug resistant (MDR) [27].

### 2.7. Detection of MRSA by Cefoxitin Disc-Diffusion Method

Susceptibility of *S. aureus* isolates to cefoxitin (30 µg) was determined by modified Kirby–Bauer disc-diffusion-method following CLSI guidelines. These trains of *S. aureus* found to be resistant to cefoxitin (isolates exhibiting zone of inhibition ≤ 21 mm in diameter) were screened as MRSA [26,28,29].

### 2.8. Detection of mecA Gene by Polymerase Chain Reaction (PCR)

*S. aureus* isolate was grown in Louria Bertani (LB) broth at 37 °C for 24 h in an orbital shaker at 120 rpm. Chromosomal DNA from *S. aureus* was extracted. Briefly, the bacterial cells were lysed with 3–5 mg/mL lysozyme in the presence of 1/10 volume of 10% sodium dodecyl sulfate (SDS) at high pH and the lysate was neutralized. Subsequent de-proteinization with 1:1 phenol: chloroform, chromosomal DNA was precipitated with ethanol by spinning at high speed and extracted DNA was stored at 4 °C.

The forward primer 5′-ACTGCTATCCACCCTCAAAC-3′ and reverse primer 5′-CTGGTGAAGTTGTAATCTGG-3′ were used for the amplification of *mec*A gene [30]. The reaction mixture for the *mec*A gene was 25 μL and consisted of 21 μL of 1X Qiagen master mix, 0.5 μL of 10 pmole primer (forward and reverse) and 3 μL of extracted DNA template. The temperature profile for PCR included initial denaturation at 94 °C for one minute of 35 cycles, annealing at 55 °C for one minute of 35 cycles for *mec*A gene, extension at 72 °C for one minute of 35 cycles and final extension at 72 °C for seven minutes. The PCR amplification products were separated by electrophoresis through 2.5% agarose gel and visualized by staining with ethidium bromide. The 163 bp amplicon was detected against 1 kb DNA ladder [31,32].

### 2.9. Quality Control

The quality of media prepared was checked by subjecting one plate of each batch for sterility and performance test. Purity plate was used to ensure that the inoculation used for the biochemical tests was pure and to see whether the biochemical tests were performed in an aseptic condition or not. For quality control, *S. aureus* ATCC 700,699 (*mec*A positive) and *S. aureus* ATCC 29,213 (*mec*A negative) were used. For PCR controls, sterile water (negative) and the known positive DNA and negative controls from previous extraction (positive) were processed for maintaining quality control.

### 2.10. Data Analysis

All the data obtained were entered into SPSS (version 22.0) and was analyzed. Descriptive as well as comparison of antibiotic susceptibility, *mec*A gene, biofilm formation was done between MRSA and MSSA.

## 3. Results

### 3.1. Growth Pattern and Distribution of Bacteria

**Figure 1:**
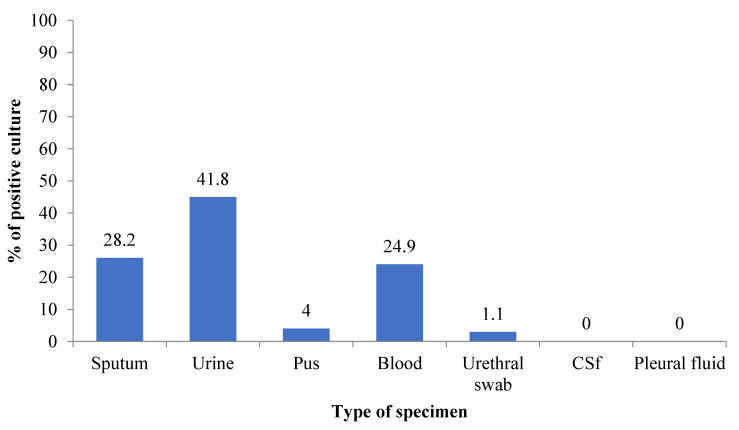
Bacterial growth status in clinical samples.

Out of 1968 clinical samples processed, growth was observed only in around 9.0% (177/1968) samples. The highest growth rate was noted in urine 41.8% (74/177) followed by sputum 28.2% (50/177), blood 24.9% (44/177) and pus 4% (7/177). No growth was observed in pleural fluid and CSF (Figure 1).

Among 177 culture positive samples, 19.2% (34/177) showed the growth of Gram-positive isolates only and 80.8% (143/177) showed the growth of Gram-negative isolates only. In the Gram-positive category, *S. aureus* 15.3% (27/177) was predominant followed by coagulase-negative *S. aureus* (CoNS) 2.8% (5/177) and *S. pneumoniae* 1.1% (2/177) whereas in the Gram-positive category, *E. coli* 38.4% (68/177) was predominant followed by *K. pneumoniae* 8.5% (15/177), *S.* Typhi 7.3% (13/177) and *P. aeruginosa* 5.6% (10/177) (Table 1).

### 3.2. Distribution of MRSA and MSSA in Different Clinical Specimens

Among 27 *S. aureus,* 15 (55.6%) were MRSA and 12 (44.4%) were MSSA. Higher number of MRSA isolates were recovered from urine 75% (3/4) followed by sputum 66.7% (4/6) and pus 66.7% (2/3) whereas greater number of MSSA isolates were observed in blood 57.1% (8/14) (Table 2).

### 3.3. Antibiotic Susceptibility Status and MDR S. aureus

Of 27 isolates of *S. aureus*, 44% were MDR. Of 15 MRSA, 5% were found to be MDR and 40% were found to be non-MDR. There was no significant association observed between MDR and methicillin resistance (*p* > 0.05). All the MRSA strains were found resistant to penicillin (*n* = 15, 100%) followed by erythromycin (*n* = 13, 86.6%). Among MSSA strain, 66.6% were resistant to erythromycin (Table 3).

### 3.4. Distribution of Biofilm Producers among MRSA and MSSA

Out of 27 *S. aureus* isolates, 1 (4%) isolate was strong biofilm producer, 19 (70%) isolates were weak biofilm producers and 7 (26%) were moderate biofilm producers. Among 19 weak biofilm producers, 52.6% were MRSA strains and 47.4% were MSSA strains. Among 7 moderate biofilm producers, 71.4% were MRSA strains and 28.6% were MSSA strain and 1 (100%) MSSA strain was strong biofilm producer (Table 4).

### 3.5. Antibiotic Susceptibility Pattern among Different Biofilm Producers

Among weak biofilm producers, 16 (84.2%) were resistant to penicillin followed by erythromycin (78.9%). Similarly, all the moderate biofilm producers were resistant to penicillin followed by cefoxitin (85.7%) and erythromycin (71.4%). Strong biofilm producer was resistant to most of the antibiotic used (Table 5).

### 3.6. Detection of mecA Gene in MRSA and MSSA Isolates

**Figure 2:**
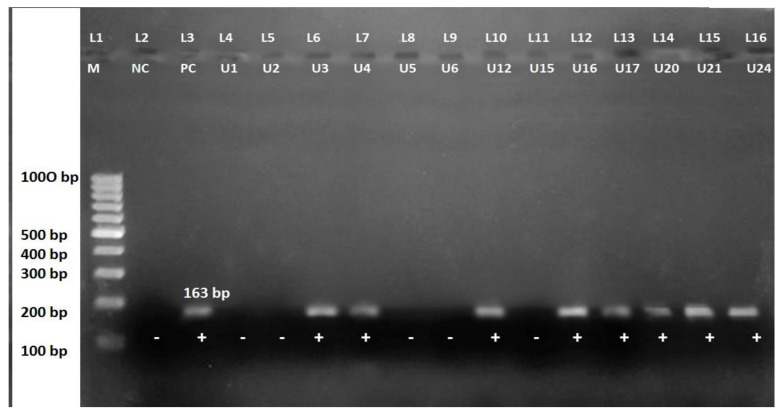
*mec*A gene amplified product after PCR in agarose gel electrophoresis. Lane 1:100 bp DNA ladder; lane 2: negative control; lane 3: positive control; lane 4: U1; lane 5: U2; lane 6: U3; lane 7: U4; lane 8: U5; lane 9: U6; lane 10: U12; lane 11: U15; lane 12: U16; lane 13: U17; lane 14: U20; lane 15: U21; lane 16: U24.

All 27 isolates of *S. aureus* including both MSSA and MRSA were selected for PCR. Among 15 MRSA, 8 isolates possessed *mec*A gene detected in 163bp (Figure 2). No *mec*A gene was detected in 12 MSSA strains.

## 4. Discussion

The current study deciphers that *S. aureus* is one of the important bacteria in various clinical infections. The proportion of MRSA was more than half among *S. aureus* isolates. Biofilm contributes to increased resistance to antibiotics. In our study, all the phenotypic MRSA had *mec*A gene but none of MSSA had *mec*A gene. Bacterial growth pattern in the present study is in concert with most of the past studies reported from Nepal, the predominant bacterial isolates identified being *E. coli* [33,34,35,36].

Among numerous samples suspected of infection, only 8.9% (177/1968) showed bacterial growth. The low growth rate might be due to inclusion of almost every patient’s sample for laboratory tests. Low percentage of growth positivity has been reported in many studies [37,38]. Among the different samples, relatively high growth rate was observed in urine compared to other specimens similar to a previous report [39].

Gram-negative isolates were almost five times more than the Gram positives in our study. *E. coli* was the most predominant Gram-negative bacterial isolate whereas *S. aureus* was the most predominant Gram-positive bacterial isolate. This result indicates that Gram-negative bacteria are emerging as important healthcare-associated pathogens [40,41]. Abundance of bacteria in environment, ability to form biofilms on many uninhabitable surfaces may have contributed to the highest prevalence of *E. coli* and *S. aureus* [42,43]. However, varying results have been reported such as *Pseudomonas* as the most predominant bacteria [37], *Acinetobacter* as the most predominant bacteria [44,45] in different hospitals.

Among 27 isolates of *S. aureus*, 55.6% isolates were screened as methicillin-resistant. Some studies reported similar prevalence of MRSA [28,46,47,48,49] whereas other studies reported high prevalence of MRSA [19,50]. The variation in the prevalence of MRSA could be due to the difference in locations and time periods of the studies, infection control measures, antibiotic prophylaxis and treatments used in each hospital and the epidemic nature of these microorganisms [51].

Prevalence of MDR *S. aureus* was found to be 44%. The overuse of antibiotics clearly drives the evolution of resistance [52]. In bacteria, antibiotic resistance occurs due to horizontal gene transfer among different species of bacteria and spontaneously through mutation [53]. Therefore, the higher prevalence may be due to haphazard use of antibiotics for treatment which is common practice in Nepal [54,55].

In our study, high percentage of both MRSA and MSSA were resistant to penicillin similar to previous studies reported from Kathmandu Medical College [56] and Nepal Medical College [11]. Following penicillin, majority of the *S. aureus* isolates were resistant to erythromycin. Erythromycin resistance may be due to its random use to cure generalized and pyogenic infections [57]. Among MRSA tetracycline was most sensitive antibiotic which echoes with a previous study from Nepal [58].

In our study, all the isolates of *S. aureus* were detected as biofilm producers. Since *S. aureus* were isolated from clinical specimens, it could be one of the reasons for high prevalence of biofilm producers. Our finding echoes with the previous studies reported from Poland (99.2%) [59] and Iran (93.1%) [60]. Majority of both the MRSA and MSSA formed weak biofilm. None of the MRSA were strong biofilm producer while the only one MSSA was a strong biofilm producer. Biofilm formation depends on many factors such as environment, availability of nutrients, geographical origin, types of specimen, surface adhesion characteristics and genetic makeup of the organism [61].

All isolated strains of *S. aureus* were selected for PCR and 53.3% MRSA isolates possessed *mecA* gene and all MSSA isolates were *mecA* negative. Similar results were obtained in the study performed by Siddiqui et al. (2018) [49] in which only 36.5% were *mecA* positive and all MSSA isolates were negative on PCR amplification.

## 5. Conclusions

The prevalence of *S. aureus* was 15.3% among different clinical specimens in a tertiary-care hospital. The current study shows a higher incidence of MRSA with most of them being multidrug resistant. More than half of the MRSA isolates possessed *mec*A gene and all the *S. aureus* isolates were biofilm producers though majority of them (70%) were weak biofilm producers. Development of antimicrobial stewardship program and regular detection of biofilm production is the need for the hour.

## 6. Strengths and Limitations

Most of the research works of similar kind conducted in Nepal barely concentrate on phenotypic characterization. The major strength of the study lies in the detection of *mec*A gene moreover observation of biofilm formation among the antibiotic resistant isolates of *S. aureus*. The findings of the study can help tertiary-care health facilities to formulate antimicrobial policies to dwindle the rate of dissemination of multidrug resistant isolates. There are few drawbacks; however, the prominent ones being a shorter timeframe of the study, a lower sample size, and the fact that it was conducted in a single hospital. Further research of this sort should investigate the genotyping expression moreover merely detecting the gene.

## Figures and Tables

**Table 1:** Growth status of Gram-positive and Gram-negative bacterial isolates (*n* = 177).

Bacteria Isolated	Gram-Positive		Gram-Negative		Total (%)
	No.	%	No.	%	
*S. aureus*	27	15.3			27 (15.3)
CoNS	5	2.8			5 (2.8)
*S. pneumoniae*	2	1.1			2 (1.1)
*E. coli*			68	38.4	68 (38.4)
*P. aeruginosa*			10	5.6	10 (5.6)
*K. pneumoniae*			15	8.5	15 (8.5)
*S*. Typhi			13	7.3	13 (7.3)
*S*. Paratyphi			6	3.4	6 (3.4)
*Enterobacter* spp			5	2.8	5 (2.8)
*B. catarrhalis*			9	5.1	9 (5.1)
*Acinetobacter* spp			3	1.7	3 (1.7)
*K. oxytoca*			5	2.8	5 (2.8)
*Providencia* spp			3	1.7	3 (1.7)
*C. freundii*			1	0.6	1 (0.6)
*H. influenzae*			1	0.6	1 (0.6)
*E. faecalis*			1	0.6	1 (0.6)
*N. gonorrhoeae*			3	1.7	3 (1.7)
Total	34	19.2	143	80.8	177 (100)

**Table 2:** Distribution of MRSA and MSSA in different clinical specimens.

Types of Specimens	MRSA (%)	MSSA (%)	Total (*S. aureus*)
Sputum (*n* = 50)	4 (66.7)	2 (33.3)	6 (22.2)
Pus (*n* = 7)	2 (66.7)	1 (33.3)	3 (11.1)
Blood (*n* = 44)	6 (42.9)	8 (57.1)	14 (51.9)
Urine (*n* = 74)	3 (75)	1 (33.3)	4 (14.8)
Total	15 (55.6)	12 (44.4)	27 (100)

**Table 3:** Antibiotic resistant pattern between MSSA and MRSA and MDR status.

Antibiotics	Type				*p*-Value
	MRSA (*n* = 15)		MSSA (*n* = 12)		
	No. of Resistant	%	No. of Resistant	%	
Tetracycline	1	6.6	2	16.7	0.068
Ciprofloxacin	5	33.3	5	41.6	
Gentamycin	4	26.7	2	16.7	
Clindamycin	4	26.7	1	8.3	
Cotrimoxazole	5	33.3	2	16.7	
Erythromycin	13	86.6	8	66.6	
Penicillin	15	100.0	9	75.0	
MDR	9	60.0	3	25.0	

**Table 4:** Distribution of biofilm producers among MRSA and MSSA.

Type of Biofilm Producer	Type of Strain		Total (%)
	MRSA (%)	MSSA (%)	
Weak biofilm producer	10 (52.6)	9 (47.4)	19 (70.4)
Moderate biofilm producer	5 (71.4)	2 (28.6)	7 (25.9)
Strong biofilm producer	0	1 (100)	1 (3.7)
Total	15	12	27 (100)

**Table 5:** Antibiotic susceptibility pattern among biofilm producers.

Antibiotics	Weak Biofilm Producers		Moderate Biofilm Producers		Strong Biofilm Producers	
	Resistant (%)	Sensitive (%)	Resistant (%)	Sensitive (%)	Resistant (%)	Sensitive (%)
Penicillin	16 (84.2)	3 (15.8)	7 (100)		1 (100)	
Cefoxitin	9 (47.4)	10 (52.6)	6 (85.7)	1 (14.3)		1 (100)
Tetracycline	1 (5.3)	18 (94.7)	1 (14.3)	6 (85.7)	1 (100)	
Ciprofloxacin	7 (36.8)	12 (63.2)	2 (28.6)	5 (71.4)	1 (100)	
Gentamycin	4 (21.1)	15 (78.9)	1 (14.3)	6 (85.7)	1 (100)	
Clindamycin	5 (26.3)	14 (73.7)	0	7 (100)		1 (100)
Cotrimoxazole	3 (15.8)	16 (84.2)	3 (42.9)	4 (57.1)	1 (100)	
Erythromycin	15 (78.9)	4 (21.1)	5 (71.4)	2 (28.6)	1 (100)	

## Data Availability

All the data related to this study are within the manuscript.
